# Semisupervised Semantic Segmentation with Mutual Correction Learning

**DOI:** 10.1155/2022/8653692

**Published:** 2022-10-03

**Authors:** Yifan Xiao, Jing Dong, Dongsheng Zhou, Pengfei Yi, Rui Liu, Xiaopeng Wei

**Affiliations:** ^1^Key Laboratory of Advanced Design and Intelligent Computing, Ministry of Education, Dalian University, Dalian 116622, China; ^2^School of Computer Science and Technology, Dalian University of Technology, Dalian 116024, China

## Abstract

The semisupervised semantic segmentation method uses unlabeled data to effectively reduce the required labeled data, and the pseudo supervision performance is greatly influenced by pseudo labels. Therefore, we propose a semisupervised semantic segmentation method based on mutual correction learning, which effectively corrects the wrong convergence direction of pseudo supervision. The well-calibrated segmentation confidence maps are generated through the multiscale feature fusion attention mechanism module. More importantly, using internal knowledge, a mutual correction mechanism based on consistency regularization is proposed to correct the convergence direction of pseudo labels during cross pseudo supervision. The multiscale feature fusion attention mechanism module and mutual correction learning improve the accuracy of the entire learning process. Experiments show that the MIoU (mean intersection over union) reaches 75.32%, 77.80%, 78.95%, and 79.16% using 1/16, 1/8, 1/4, and 1/2 labeled data on PASCAL VOC 2012. The results show that the new approach achieves an advanced level.

## 1. Introduction

As a fundamental task, semantic segmentation is widely used in medical image diagnosis [1], automatic driving [2], and other fields, which is the process of defining the boundaries between the various semantic entities in an image. From a technical point of view, each pixel in the image is assigned a category or semantic label. With the development of deep learning, fully supervised semantic segmentations [3–7] achieve success, but they all need enough pixel-level labels to complete the representation learning, which requires a lot of manpower.

Weakly supervised and semisupervised semantic segmentation effectively reduces the annotation burden. Weakly supervised methods use weak annotations as labels to train segmentation models. Semisupervised methods combine additional unlabeled data with a small amount of labeled data to improve segmentation model performance and close the gap with supervised models trained from fully pixel-labeled data. How to use unlabeled data for training models to get good segmentation performance is a problem we need to solve.

In semisupervised semantic segmentation, the methods are mainly based on adversarial learning [8–10] and consistency regularization [11, 12]. The generative adversarial network (GAN)-based approach [8] proposed a full convolution discriminator, which can learn to distinguish the ground truth and the output of the generator, enhancing the consistency between the predicted maps of the segmentation network and the ground truth. Consistency regularization enforces the prediction consistency of perturbations by increasing the input image perturbation [11, 12], the feature perturbation [13], and the network perturbation [14] to make the prediction consistent among the output of multiple perturbations. Chen et al. [15] proposed the cross pseudo supervision loss, in which unlabeled data were input into two segmentation networks with different initializations to generate pseudo labels for cross supervision and strengthen the consistency of the model.

However, the cross pseudo supervision still has two drawbacks. First, the segmentation network generates inaccurate pseudo labels to guide model learning, which damages the model accuracy, and pseudo labels are directly generated by the confidence segmentation maps of unlabeled images, completely ignoring the ability of the network itself to improve pseudo labels. Second, the cross pseudo supervision is plagued by confirmation bias and tends to overfitting pseudo labels that are incorrectly predicted. After one segmentation network predicts the wrong label output, the cross pseudo supervision trains the other model with wrong knowledge, thus hindering the cross learning of the model.

To address the above two problems, we propose a new semisupervised semantic segmentation method based on cross pseudo supervision. Many works combine consistency regularization with pseudo labels, our proposed method also includes pseudo labels [16–18] and utilizes pseudo segmentation maps to enhance consistency. To address the first problem, we introduce the multiscale feature fusion attention mechanism module [19] to generate well-calibrated segmentation confidence maps, and the multiscale feature fusion attention mechanism mode fuses high-level feature maps and low-level feature maps to generate segmentation confidence maps with higher quality. To address the second problem, we propose mutual correction learning to improve the model convergence in the wrong direction caused by pseudo labels. The mutual correction loss uses the internal knowledge of pseudo labels for mutual correction, which not only strengthens the consistency of the network but also corrects the learning direction of the model. In this way, the segmentation performance of consistency training is greatly improved. To sum up, our two-fold contributions are as follows:

- We propose an effective module to generate better quality segmentation confidence maps by fusing low-level texture information and high-level semantic information of the features.
- We propose mutual correction learning for semisupervised semantic segmentation, which uses the intrinsic knowledge to correct the convergence direction of the model and effectively ameliorates the problem of model performance degradation by erroneous cross pseudo supervision.

The rest of this article is arranged as follows: The second section introduces the related work of semisupervised semantic segmentation. In the approach section, we describe the details of mutual correction learning with pseudo labels. The experimental details and results are presented in the experiment section. In the conclusion section, we summarize this paper.

### 1.1. Related Work

#### 1.1.1. Fully Supervised Semantic Segmentation

Fully convolutional networks (FCNs) [3] can accept input images of any size, and the deconvolution layer is used to perform upsampling of the feature map of the last convolution layer and predict each pixel. Although high-level features contain rich semantic information, they cannot capture long-term relationships well. Therefore, global pooling [4], dilated convolution [5], pyramid pooling [6], and attention mechanisms [7] are used to better aggregate context. Deeplabv3+ [20] fuses features of different scales to refine the object boundaries of the segmentation results. However, training supervised segmentation networks requires a large amount of labeled data, which is expensive to collect. Our work alleviates the constraints of annotated data by making efficient use of unlabeled data. To make a fair comparison with previous works, we use Deeplabv3+ as the backbone architecture.

#### 1.1.2. Weakly Supervised Semantic Segmentation

Weakly supervision is to further reduce the cost of data annotation based on full supervision. Some early works use weak annotations such as bounding boxes [21–23], scribbles [24], and image-level labels [25–28]. The recent methods use object location information to generate pseudo pixel annotations and train the segmentation network, and their segmentation performance is significantly improved. Al-Huda et al. [26] fused activation maps and saliency maps to guide the model to generate initial pixel-level annotations and generate more accurate pixel labeling through iteration. Although promising results have been obtained using the above methods, most of them require additional training strategies. Al-Huda et al. [28] proposed a new postprocessing method, which learned the concept of the object scale from the intermediate features of hierarchical structure through dynamic programming and further improved the segmentation accuracy.

#### 1.1.3. Semisupervised Semantic Segmentation

The semisupervised method is based on incomplete supervised learning, using partially labeled data and unlabeled data for model training. The semisupervised semantic segmentation method is mainly based on the idea of consistent regularization and pseudo labeling.

Consistency regularization enforces the model to make consistent predictions concerning various perturbations. Its effectiveness is based on the smoothing assumption or the cluster assumption. These assumptions consider that data pointing close to each other are likely from the same class, which are often used in classification tasks [29, 30]. As for semantic segmentation tasks, French and Ouali found that semantic segmentation tasks do not fully comply with the clustering assumption in [11, 13]. Therefore, Ouali et al. [13] proposed to perturb the output of the encoder while maintaining the clustering assumption and used multiple auxiliary decoders to obtain a consistent prediction. French et al. [11] found that mask-based enhancement strategies were effective and introduced data enhancement technology CutMix [31]. The idea of CutMix is to mix samples by replacing part region of the image with a patch from another image and treat it as an extension of Cutout [32] and Mixup [33]. Cross consistency training (CCT) [13] used shared encoders and multiple decoders as segmentation networks, and the prediction using different decoders enhanced consistency. Mittal et al. [9] proposed a dual-branch method for semisupervised semantic segmentation, the GAN-based model solved the inaccuracy of low-level details, and the semisupervised multilabel classification model corrected the misunderstanding of high-level information. Lai et al. [34] proposed different contexts in the same area to enhance the consistency of context awareness. Guided collaborative training (GCT) [35] further used different initialization segmentation networks to enhance the consistency of disturbed network prediction. Our approach combines the ideas of CutMix [31] and cross pseudo supervision (CPS) [15] to enhance the consistency between mixed output and mixed input prediction.

Pseudo labeling is a technique that utilizes unlabeled data through feature learning and alternating pseudo label prediction [16–18]. Its main goal is entropy minimization, and it encourages the network to make confident predictions of unlabeled images and prevents features from being trained to the wrong class. Chen et al. [17] proposed a new two-branch network in which the pseudo network extracted the correct pseudo labels as auxiliary supervised information for the training segmentation network. Zhou et al. [18] proposed a pseudo label enhancement strategy to improve the quality of pseudo labels. The key to pseudo labeling is the quality of pseudo labels. Most models [36, 37] refine pseudo labels from external guidance, such as teachers. However, the teacher model is often fixed, making the student inherit some inaccurate predictions from the teacher. In order to generate better pseudo labels, the recent approach is to update both the teacher and student models, such as coteaching [38], dual students [14], and metapseudo labels [39]. Furthermore, it is essential that the model converges in the right direction at the beginning of training. In the third section, mutual correction learning is used to correct the convergence direction of the model.

### 1.2. Approach

Semisupervised semantic segmentation uses labeled images *D*_*l*_ = {*x*_*l*_, *y*^*∗*^} and unlabeled images *D*_*u*_ = {*x*_*u*_} to learn a segmentation network. *x* ∈ *R*^*H*×*W*×3^ denotes the images with a resolution of *H* × *W*, *y*^*∗*^ ∈ *R*^*H*×*W*×K^ is the ground truth corresponding to *x*_*l*_ with pixels labeled by *K*classes, and*f*is a segmentation network with a weight of *θ*.

**Figure 1:**
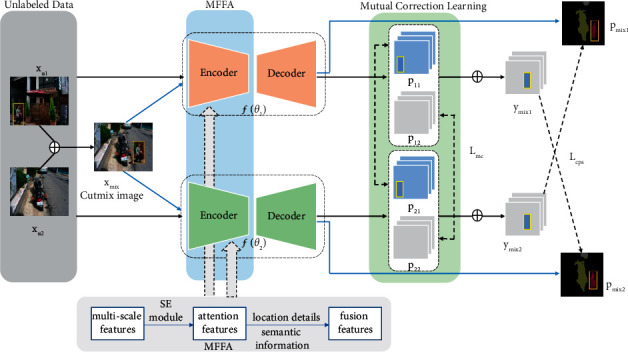
Overview of mutual correction learning. Two images *x*_*u*1_ and *x*_*u*2_ are sampled from the unlabeled dataset. The CutMix images are generated by two source images, and they are all inputted into each segmentation network. *p*_*i*1_ and *p*_*i*2_ are mixed as pseudo segmentation maps *y*_mix*i*_ to supervise the other segmentation network. ⊕: CutMix, MFFA: multiscale feature fusion attention mechanism module, *ℒ*_*mc*_: mutual correction loss, *ℒ*_*cps*_: cross pseudo supervision loss, *p*: segmentation confidence map, *y*_mix*i*_: predicted one-hot label map, and SE module: squeeze-and-excitation module.

The approach proposed in the paper is shown in Figure 1. The mutual correction learning model consists of two parallel segmentation networks. *f*(*θ*_1_) and *f*(*θ*_2_) are the same segmentation networks with different initialization. The network inputs are *x*_*u*1_, *x*_*u*2_, and *x*_mix_, unlabeled images *x*_*u*1_ and *x*_*u*2_ are with the same augmentation, and *x*_mix_ is obtained through CutMix [31] by (1), where *M* ∈ (0,1)^*W*×*H*^ is binary coding and represents the position of removing and filling from two images:

(1) $$\begin{array}{c} x_{mix}=M⊙x_{u1}+\left(1-M\right)⊙x_{u2}. \end{array}$$

*p* is the segmentation confidence map obtained after softmax normalization. The output structure with a weight *θ*_2_ is the same as the output with *θ*_1_.

(2) $$\begin{array}{c} p_{11}=f\left(x_{u1};\;\theta_{1}\right), \end{array}$$

(3) $$\begin{array}{c} p_{12}=f\left(x_{u2};\;\theta_{1}\right), \end{array}$$

(4) $$\begin{array}{c} p_{mix1}=f\left(x_{mix};\;\theta_{1}\right), \end{array}$$

*y* is the predicted pseudo label. At each position *i*, the pseudo label *y* is the one-hot map computed by the segmentation confidence map *p*, and the value of *M* in (5) is the same as that in Eq. (1).

(5) $$\begin{array}{c} y_{mix1}=M⊙p_{11}+\left(1-M\right)⊙p_{12}. \end{array}$$

#### 1.2.1. Multiscale Feature Fusion Attention Mechanism Module

Since generating pseudo labels with rich semantic information requires multiple convolution operations to continuously extract features, the dimension of features continues to expand, resulting in serious high-dimensional information redundancy. When all channel features are fused, the importance of features in each channel is not considered. Hence, Hu et al. [19] proposed the squeeze-and-excitation (SE) module for the adaptive fusion of channel features to reduce the redundancy of high-dimensional features.

**Figure 2:**
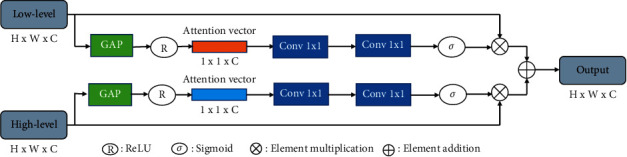
MFFA: multiscale feature fusion attention mechanism module. *σ*: sigmoid, ⊗: element multiplication, and ⊕: element addition.

This paper introduces the multiscale feature fusion attention mechanism module to fuse high-level and low-level feature maps. The attention mechanism uses two SE modules to extract different attention features from low-level features to high-level features, as shown in Figure 2. The module contains location details in low-level features and semantic information in high-level features to improve the accuracy of the prediction of different target boundaries.

In (6), the role of the global mean pooling (GAP) layer is to integrate global spatial information. It takes the feature map as input to obtain a feature vector containing semantic correlation. The attention vector is obtained by (7), and the output $\hat{x}$ of the encoder is generated by eq (8).

(6) $$\begin{array}{c} g\left(x_{k}\right)=\frac{1}{W\times H}\overset{H}{\underset{i=1}{\sum }}\overset{W}{\underset{j=1}{\sum }}x_{k}\left(i,j\right)\text{,} \end{array}$$

where *k* = 1,2,3 ⋯ *d*, *d*is channel dimensions, and *x*_*k*_is the channel input of the module.

(7) $$\begin{array}{c} A_{c}=\delta_{2}\left[\delta_{1}\left[g\left(x\right)+b_{\alpha }\right]+b_{\beta }\right]\text{.} \end{array}$$

*x* = [*x*_1_, *x*_2_, ⋯, *x*_*d*_], *g* is the GAP layer, *δ*_1_ and *δ*_2_ are activation functions ReLU and sigmoid, respectively, and *b*_*α*_ and *b*_*β*_ are the bias.

(8) $$\begin{array}{c} \hat{x}=A_{c}⊗x. \end{array}$$

The output of the encoder is the sum of low-level ${\hat{x}}_{l}$ and high-level ${\hat{x}}_{h}$, which is decoded to obtain the segmentation confidence map.

#### 1.2.2. Mutual Correction Learning

The two segmentation networks have different learning capabilities after different initialization, and they can learn online from the pseudo labels generated by each other. In the training process, if the segmentation network *f*(*θ*_1_) generates poor quality one-hot labels *y*_mix1_, the segmentation network *f*(*θ*_2_) produces a good quality confidence map *p*_mix2_, and the model may converge in the wrong direction guided by the poor quality label; the self-correction ability of the cross pseudo supervision is limited, which may degrade the performance of the model.

In order to prevent the model from converging in the wrong direction, we propose the mutual correction loss to correct this problem, and the training objectives include three losses: supervision loss *ℒ*_*s*_, mutual correction loss *ℒ*_*mc*_, and cross pseudo supervision loss *ℒ*_*cps*_. The supervision loss is not marked in the network structure diagram.

*ℒ*_*s*_: the labeled image *x*_*l*_ does not require CutMix and is input into the two networks for supervised learning. The supervision loss *ℒ*_*s*_ can be written as follows:

(9) $$\begin{array}{c} L_{s}=\frac{1}{\left|N_{l}\right|}\underset{D_{l}}{\sum }\left(1/W\times H\right)\overset{W\times H}{\underset{i=1}{\sum }}\left(l_{ce}\left(p_{1}^{i},y_{1}^{*i}\right)+l_{ce}\left(p_{2}^{i},y_{2}^{*i}\right)\right). \end{array}$$

*N*_*l*_ represents the number of labeled images in a batch, and *W* and *H* represent the width and height of the input image. *ℓ*_*ce*_ is the standard cross entropy loss function and *y*_1_^*∗i*^(*y*_2_^*∗i*^) is the ground truth.

*ℒ*_*mc*_: we propose a mutual correction loss *ℒ*_*mc*_ to make the model have the ability to self-correct. Unlabeled images *x*_*u*1_ and *x*_*u*2_ are input to the network *f*(*θ*_1_) and *f*(*θ*_2_), respectively, to produce the corresponding confidence maps *p*_11_, *p*_12_ and *p*_21_, *p*_22_. Cross entropy describes the difficulty of expressing probability distributions *p*_11_ (*p*_12_) through probability distributions *p*_21_ (*p*_22_). The smaller the value of cross entropy is, the closer the two probability distributions are. According to the consistency principle, the confidence map similarity of *p*_11_ and *p*_21_ should be higher. In other words, the loss between (*p*_11_, *p*_21_) and (*p*_12_, *p*_22_) should be as small as possible, so the mutual correction loss *ℒ*_*mc*_ can be written in the following form:

(10) $$\begin{array}{c} L_{mc}=\frac{1}{\left|N_{u}\right|}\underset{D_{u}}{\sum }\left(1/W\times H\right)\overset{W\times H}{\underset{i=1}{\sum }}\left(l_{ce}\left(p_{11}^{i},p_{21}^{i}\right)+l_{ce}\left(p_{12}^{i},p_{22}^{i}\right)\right). \end{array}$$

*ℒ*_*cps*_ [15]: The cross pseudo supervision loss is symmetric, and the pseudo label *y*_*mix*1_ is used to supervise the confidence map *p*_mix2_ generated by another network, and the other one uses the pseudo label *y*_mix2_ to supervise the confidence map *p*_mix1_. The cross pseudo supervision loss *ℒ*_*cps*_ can be written in the following form:

(11) $$\begin{array}{c} L_{cps}=\frac{1}{\left|N_{u}\right|}\underset{D_{u}}{\sum }\left(1/W\times H\right)\overset{W\times H}{\underset{i=1}{\sum }}\left(l_{ce}\left(p_{mix1}^{i},y_{mix2}^{i}\right)+l_{ce}\left(p_{mix2}^{i},y_{mix1}^{i}\right)\right). \end{array}$$

When training the segmentation network, we use multiple loss constraints on the segmentation network and minimize them for tuning. *γ* and *λ* are the hyperparameters set by the experiment, and the loss function of the whole training can be written as follows:

(12) $$\begin{array}{c} L=L_{s}+\gamma L_{mc}+\lambda L_{cps}. \end{array}$$

### 1.3. Experiments

#### 1.3.1. Datasets

PASCAL VOC 2012 [40] is the most widely used benchmark dataset for semantic segmentation tasks. Pascal VOC 2012 training set used in this paper contains 10,582 images and annotations, and the validation set contains 1449 images and annotations. PASCAL VOC has a total of 20 categories, such as aircraft, bicycles, birds, and boats.

Cityscapes [41] contains tagged images of urban street scenes taken from vehicles driven in European cities, specifically for semantic understanding of urban street scenes. It has 19 category tags and contains 5000 finely labeled images, including 2975 images for network training, 500 images for network verification, and 1525 images for testing. In addition, we only used the fine annotation graph for training.

Following the division protocol of GCT [35], the entire training set was randomly divided into two groups, with 1/2 (5291), 1/4 (2646), 1/8 (1323), and 1/16 (662) of the whole training set as the labeled set.

#### 1.3.2. Evaluation Metrics

Mean intersection over union (MIoU) is a common evaluation metric in semantic segmentation. In (13), where *TP*_*c*_, *FP*_*c*_, and *FN*_*c*_ represent the prediction results of true positive, false positive, and false negative of category *c*, *C* represents the total number of categories.

(13) $$\begin{array}{c} \text{MIoU}=\frac{1}{C}\overset{C}{\underset{c=1}{\sum }}\left(TP_{c}/TP_{c}+FP_{c}+FN_{c}\right). \end{array}$$

For all experiments, we used only one network for inferential prediction, testing the results of the 1456 PASCAL VOC 2012 value set (or 500 Cityscapes value set).

#### 1.3.3. Implementation Details

The PyTorch deep learning framework was used to complete the proposed method and related experiments. We used ResNet-101 pretrained on ImageNet as backbone and SyncBN [42] for training. Our method set weight decay as 0.0005 and momentum as 0.9. The loss weights *γ* and *λ* are 1 and 1.5 on PASCAL VOC and 1.5 and 6 on cityscapes. We used a multiple learning rate strategy, and the initial learning rate values were set to 0.0025 for PASCAL VOC, while 0.02 for Cityscapes.

#### 1.3.4. Comparison with Other Methods

**Figure 3:**
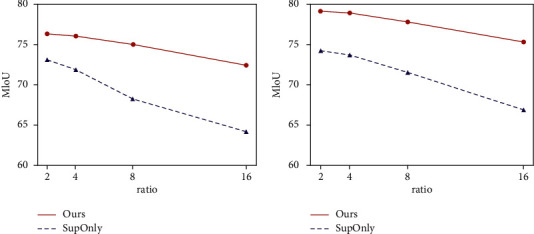
Comparison with SupOnly in PASCAL VOC 2012 (1/2, 1/4, 1/8, 1/16). (a) ResNet-50. (b) ResNet-101.

In Figure 3, the improvements of this method are shown under different label proportions. All methods are based on DeepLabv3+.

Figure 3(a) shows that our approach using ResNet-50 consistently outperforms the supervised baseline approach on PASCAL VOC 2012. The improvements of our method over the baseline method are 8.28%, 6.80%, 4.23% and 3.28% under 1/16, 1/8, 1/4, and 1/2 scale settings separately.  Figure 3(b) shows that our method uses ResNet-101 for 8.45%, 6.26%, 5.26%, and 4.94% lift at 1/16, 1/8, 1/4, and 1/2 scale settings, respectively.

We compared our method with some recent semisupervised segmentation methods, including cross consistency training (CCT) [13], guided collaborative training (GCT) [35], context-aware consistency (CAC) [34], and cross pseudo supervision (CPS) [15] under different segmentation protocols.  Table 1 shows the experimental comparison results on PASCAL VOC 2012. In different scale settings, our method is superior to other methods, whether ResNet-50 or ResNet-101. Especially in 1/8 and 1/4 proportions, it was 1.43% and 1.20% and 1.36% and 1.27% higher than CPS, respectively.

We further verified the effectiveness of the proposed method by comparing with other methods on Cityscapes in Table 2. Compared with CCT and GCT, the accuracy of our method is greatly improved with a small number of labeled images, especially in the case of 1/16. The main reason for the low improvement on Cityscapes results compared to PASCAL VOC 2012 is that PASCAL VOC 2012 is an object-centered semantically segmentation dataset with an average of three objects per image. Cityscapes is a highly complex urban street scene, and the resolution and scene complexity of each picture are much higher than those of PASCAL VOC 2012, which will lead to the inclusion of more complex information in the mutual correction learning and weaken the ability of mutual correction. Therefore, our method is more suitable for each dataset with fewer graph object instances.

#### 1.3.5. Ablation Study

The ablation study in Table 3 shows the contribution of each function. The ablation study was based on PASCAL VOC 2012 with 1/8 labeled data. DeepLabv3+ and ResNet-50 were the segmentation networks. The supervised loss training (SupOnly) model was used as the benchmark of our work.

In Table 3, ID 2 shows the performance improvement with cross pseudo supervision losses, with 5.37% MIoU improvement on PASCAL VOC 2012 compared to ID 1 with supervised losses alone.

In order to prove the validity of the multiscale feature fusion attention mechanism module, we made a comparison between the model with MFFA and the model with the cross pseudo supervised loss. Features of different scales combine rich localization and semantic information to generate accurate segmentation maps of boundary information. ID 2 and ID 3 showed that the model with the MFFA module improved by 0.36% compared with the model with cross pseudo supervision loss. In addition, ID 4 and ID 5 found that MFFA improved by 0.82%.

In ID 2 and ID 4, the effectiveness of the mutual correction loss was compared with that of the supervised loss and cross pseudo supervised loss. The cross pseudo supervision learns the error information and corrects it effectively through the mutual correction loss, and MIoU shows an increase of 0.61%. ID 3 and ID 5 found that the mutual correction loss increased MIoU by 1.07% while using the MFFA module. According to ID 5, MIoU improved by 1.43% with the multiscale feature fusion attention mechanism module and mutual correction loss.

#### 1.3.6. Qualitative Results

**Figure 4:**
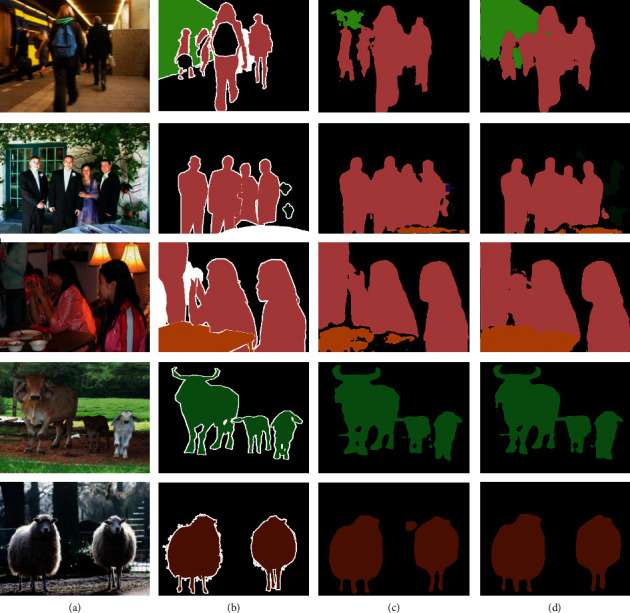
Example qualitative results from PASCAL VOC 2012. All the approaches are trained under 1/8 with ResNet-101 as the backbone: (a) input; (b) ground truth; (c) CPS; (d) ours.

Figure 4 shows the results of different methods on PASCAL VOC 2012. The actual labels are shown in column (b), CPS (column (c)), and predicted boundary errors, and our method corrects these problems in column (d). Obviously, mutual correction learning can more accurately predict the edges and categories of objects, thus improving the feature representation of the model.

#### 1.3.7. Limitations

When the output predictions of the two segmentation networks are both wrong, the error correction of the mutual correction learning is limited. The results also show that our approach is influenced by the distribution of long-tail classes on semantic segmentation datasets, which makes pseudo labels biased towards majority classes, and we will continue to study it and improve further.

## 2. Conclusion

We propose a semisupervised semantic segmentation approach based on mutual correction learning. The MFFA module is introduced to generate confidence maps, which in turn yield well-calibrated pseudo labels. To alleviate the problem of poor quality pseudo labels guiding the model to learn misinformation, we propose a mutual correction loss, utilizing the internal knowledge to correct the convergence direction of the model. Experiments show our approach further narrows the gap between fully supervised and semisupervised semantic segmentation.

## Figures and Tables

**Table 1:** Comparison with other methods on PASCAL VOC 2012 under different partition protocols. The segmentation network is DeepLabv3+. SupOnly represents supervised training, using only labeled data.

Method	ResNet-50			
	1/16 (662)	1/8 (1323)	1/4 (2646)	1/2 (5291)
SupOnly	64.20	68.30	71.87	73.12
CCT [13]	65.22	70.87	73.43	74.75
GCT [35]	64.05	70.47	73.45	75.20
CAC [34]	70.10	72.40	74.00	76.50
CPS [15]	71.98	73.67	74.90	76.15
Ours	**72.48**	**75.10**	**76.10**	**76.40**

Method	ResNet-101			
	1/16 (662)	1/8 (1323)	1/4 (2646)	1/2 (5291)
SupOnly	66.87	71.54	73.69	74.22
CCT [13]	67.94	73.00	76.17	77.56
GCT [35]	69.77	73.30	75.25	77.14
CAC [34]	72.40	74.60	76.30	78.20
CPS [15]	74.48	76.44	77.68	78.64
Ours	**75.32**	**77.80**	**78.95**	**79.16**

The meaning of the bold values represent the best results.

**Table 2:** Comparison with other methods on Cityscapes under different partition protocols. The segmentation network is DeepLabv3+, and the backbone is ResNet-50. SupOnly represents supervised training, using only labeled data.

Method	ResNet-50			
	1/16 (186)	1/8 (372)	1/4 (744)	1/2 (1488)
CCT [13]	66.35	72.46	75.68	76.78
GCT [35]	65.81	71.33	75.30	77.09
CAC [34]	—	69.70	72.70	—
CPS [15]	74.47	76.61	77.83	78.77
Ours	**74.47**	**76.75**	**78.03**	**78.89**

The meaning of the bold values represent the best results.

**Table 3:** Ablation study. *ℒ*_*s*_: supervised loss. *ℒ*_*cps*_: cross pseudo supervised loss. *MFFA*: multiscale feature fusion attention mechanism module. *ℒ*_*mc*_: mutual correction loss.

ID	*ℒ*_*s*_	*ℒ*_*cps*_	*MFFA*	*ℒ*_*mc*_	MIoU
1	✓				68.30
2	✓	✓			73.67
3	✓	✓	✓		74.03
4	✓	✓		✓	74.28
5	✓	✓	✓	✓	75.10

## Data Availability

Previously reported PASCAL VOC 2012 and Cityscapes datasets were used to support this study and are available at DOI: https://doi.org/10.1007/s11263-014-0733-5 and DOI: https://doi.org/10.1109/cvpr.2016.350. These prior studies (and datasets) are cited at relevant places within the text as references [40, 41].
